# A systemic C balance as a tool to evaluate the environmental role of complex and integrated productive systems

**DOI:** 10.1016/j.mex.2024.103132

**Published:** 2024-12-22

**Authors:** Davide Biagini

**Affiliations:** Dipartimento di Scienze Agrarie, Forestali e Alimentari, Università di Torino, Largo Paolo Braccini 2, 10095 Grugliasco (TO), Italy

**Keywords:** Carbon balance, Carbon fluxes, Carbon storage, Resources depletion, Resources enrichment, Systemic C balance

## Abstract

The most frequently used environmental assessment methods are often applied, through a product-based approach, to analyse a single productive process, but such methods neglect the interactions that characterise complex and integrated biological systems (CIS), and often only consider the negative impacts. In order to overcome these gaps and since a material balance can highlight the relationships and interactions of a CIS, a systemic carbon (C) balance is here proposed as a complementary analysis tool to highlight the relationships that exist between different productive processes carried out by the same production unit and to characterise its environmental role. The method was applied to a beef farm consortium to its validation. Conducting a mass balance involves considering the input and output material flows and their accumulation within a system. The systemic C balance applied to the consortium was found to result in the removal of 96,100 t C from the atmosphere over a period of one year, thereby leading to different conclusions from those obtained with other assessment methods. Based on these results, it appears that here presented method can describe a CIS and to highlight the relationship that exists between rearing and agricultural activities in order to characterise their environmental roles.

• A new approach to CIS evaluation is presented.
• C fluxes were used to evaluate the relationships among integrated productive processes.
• A mass balance can be used to evaluate resource depletion or the enrichment of a system.

## Graphical abstract

**Figure:**
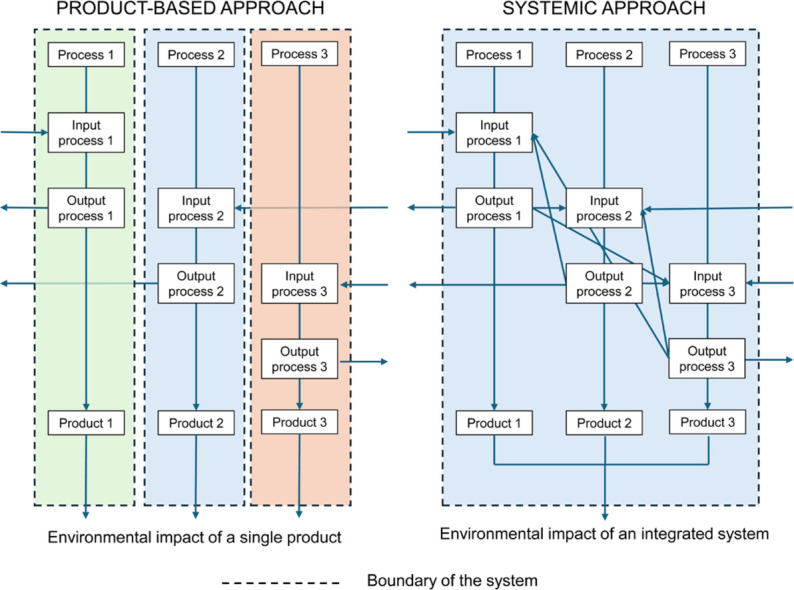

Specifications table

**Table:** 

Subject area:	Environmental Science
More specific subject area:	Agricultural and Animal Sciences
Name of your method:	Systemic C balance
Name and reference of original method:	**Material balance for ecological system**. Odum, H.T., 1956. Primary production in flowing waters. Limnol. Oceanogr. 1, 102–117. https://doi.org/10.4319/lo.1956.1.2.0102.
Resource availability:	Not applicable

## Background

A complex system is defined as an entity that has many components that interact closely with each other in an integrated manner [1]. A livestock farm is an example of a complex and integrated production system because its activities, especially those pertaining to cattle, are closely linked to the cultivation activities, not only to the production of forages and crops to feed animals but also, through the spreading of manure on fields, to those not specifically intended for livestock feed production. These relationships are often not considered in the most frequently used assessment methods, which focus on the single production process of a product or a service. Such methods often analyse the entire life cycle, through a product-based approach, but do not consider the services associated with the production and neglect the interactions that take place in a complex system. Furthermore, they generally focus on the negative aspects of an environmental impact and neglect the positive ones, or consider them separately, and they sometimes even tend to misrepresent less intensive agriculture production systems [2].

Since the relationships and interactions that take place in the productive processes carried out by a company can affect the material balance of a system that undergoes the depletion of resources through a productive process, but with the possible preservation or recovery by another one, a method has been proposed in this study to draw up a systemic carbon (C) balance in order to estimate the C fluxes and the changes in its stocks in the system. A mass balance, by considering the farm as a whole and not just as a production process, is able to assess the real impact of a multi-functional production activity and, despite its limitations, can offer a clearer vision of the interactions that take place within a complex system, such as a livestock farm. The principle of the material and energy balance was introduced, at an ecological system level, by Odum [3]. Woodwell and Whittaker [4], who worked on nutrient cycling, C storage, and ecosystem productivity, contributed to the development of mass balance concepts. The mass balance, if applied to a productive system, requires a correct identification of all the relationships and the C input and output pathways. This approach has been widely used over the last sixty years in agricultural and livestock systems to evaluate the management of nutrients, crop residues and manure [5], to evaluate the feed efficiency and nutrient excretion of livestock [6] and to evaluate sustainable practices [7], albeit through different approaches, according to the aims of the research or without fully representing the farm level C balance, since they do not address all the C input and output pathways. For example, some trials applied the C balance to single productive processes, such as intensive pasture [8] or grazing lands [9], without considering the production systems to which they belong. Others did not consider all the C pathways, that is, the release of respiratory gases [10], farmyard emissions and supplementary feeds given to the animals [11] or they did not include enteric emissions from cattle [12].

The systemic C balance presented in this paper can be seen as an extension of the original approach proposed by Odum [3] to remedy the aforementioned gaps and adapted to a productive system to obtain indicators that are useful for its characterisation.

## Method details

A systemic C balance is hereafter described and exemplified for the case of a mammalian livestock farm in order to highlight, through the use of a new tool, the environmental roles of a productive system that are often misrepresented by other assessment methods and to compare the performances of such systems. The methodology involves the following steps.

Step 1, definition of the boundaries of the system.

Description: such a definition is useful to identify what processes and stages can be carried out by the system, and the ones that cannot.

Example: the boundaries of a specialised growing-finishing cattle landless livestock production system only include the fattening process, but the boundaries on a generic multipurpose whole-cycle livestock farm could include the productive processes that are based on animal, plant and energy production, including the transformation of the obtained raw products (milk, meat, grain, fruits, gases, etc.). Moreover, a consortium of livestock farms cold be considered as a single system.

Step 2, identification of the productive processes involved in the system and their relationships.

Description: an integrated production system is a system that combines different productive processes in which the outputs of one productive process (products, by-products or wastes) represent the inputs of another process. The exchange of materials among different productive processes can be explicit and therefore easy to identify, or implicit and therefore more difficult to determine. Each relationship should be considered.

Example: the processes carried out on a generic whole-cycle livestock farm may be based on the management of animals on permanent grasslands (related to livestock), the cultivation of annual or pluriannual species devoted to producing feeds (related to livestock), the cultivation of annual or pluriannual species devoted to the market (related to livestock for some by-products or to raw product transformation for the farm), the cultivation of non-harvested species (related to insect livestock, e.g. bees), wood production (related to energy production), other land for different uses (related to energy production), animal reproduction (related to animal fattening, the cultivation of annual or pluriannual species and energy production), animal fattening (related to breeding animals, annual or pluriannual species and energy production), the transformation of raw products or by-products of animal or vegetable origin on the farm (related to crops, livestock or energy production), and energy production (related to several productive processes carried out on the farm).

Step 3, graphic representation of the identified connections (flowchart).

Description: the relationships that exist among the productive processes identified in step 2 are drawn up using arrows or boxes to represent internal and external material fluxes and relevant stocking sites, respectively.

**Fig. 1:**
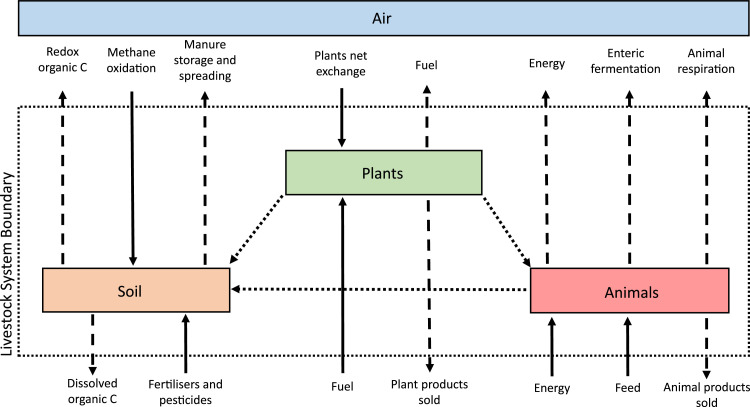
Schematic representation of the C fluxes and the considered storage sites. The inputs are represented by solid arrows, the outputs by broken arrows, the internal fluxes by dotted arrows, while the squares represent the storage sites.

Example: Fig. 1 graphically represents the inputs, outputs and internal fluxes as well as the connections among the stocking sites of a livestock farm.

Step 4, collection of raw data.

Description: all the data that are useful for quantifying the flow of materials should be measured directly, estimated, calculated or collected from official documentation and then completed and verified through site visits.

Example: the raw data pertaining to a cattle farm that have to be collected, with reference to the utilisation of the farm surfaces, the cultivated species, field areas, the average crop and wood production yields, the used productive factors (self-produced or purchased), the cultivation operations, the sold productions, categories and number of reared animals, the average live-weight of the heads, the composition of the diets, feed dry matter intake, the average number of parities per cow per year, the calving interval, the average milk produced per cow per year, the fat and protein contents of the milk, the growth rate of the heads, the number, age, weight and categories of the animals slaughtered per year, and the energy sources and consumptions.

Step 5, aggregation of the collected data (if appropriate).

Description: homogeneous data belonging to different productive processes can be aggregated to simplify the subsequent steps.

Example: the weight gain of fattening and breed animals or the crop/grassland area of different productive units in a consortium can be grouped together and added.

Step 6, calculation of the derived data and evaluation of their most probable range of variation.

Description: the derived data are calculated with mathematical models, formulas, coefficients, etc. starting from the raw data. It is possible to estimate the most probable range of variation of the variables by referring to bibliographic references or to information obtained from knowledge on the specific rate of sensitivity of these variables evaluated during the direct collection of the data

Example: the C net exchange of plants on a livestock farm can be calculated (from coefficients, if not measured directly, for example with the Eddy Covariance technique, which, however, measures the net ecosystem exchange – NEE – that considers the respiration of the ecosystem and therefore already includes the depletion of the organic matter in the soil); crop residues (from the coefficients); body composition of the animals (from the coefficients or bibliographic data); respiratory gas exchange (from formulas or direct/indirect measurements); enteric gas production (from formulas or direct/indirect measurements); faeces and urine excretion (from formulas, mathematical models or direct measurements); manure spreading (from mathematical models, coefficients or direct measurements); fuels consumed per farm operation (from formulas or coefficients); dissolved organic C (from formulas or coefficients); organic matter depletion for oxidation or reduction (from coefficients or direct measurements, if not already considered in the NEE), and methane oxidation by soils (from coefficients). Considering how difficult it is to correctly measure enteric gases, an attempt was made to obtain them as the difference between the C input (feed), C retention (average weight gain) and C outputs (milk, calves, breathing, faeces and urine) in order to consider the production of both carbon dioxide and methane. This approach would formally be more correct than the direct measurement of enteric gases, because many of the techniques adopted for this purpose (e.g. SF_6_ tracer gas, or open-path laser devices) only detect enteric methane and not carbon dioxide. The direct collection of raw data allows the rate of sensitivity of the variables involved in the balance, e.g. the range of variation of the crop yields or the slaughtering weight of the animals to be evaluated by means of descriptive statistic tools. However, it is possible to find the range of variation of the variables of the derived data that have not been measured directly in the literature, e.g. it is possible to apply the range of variability published in the reports of the Intergovernmental Panel on Climate Change for methane emissions due to rumen fermentation.

Step 7, transformation of the material and energy fluxes into C fluxes.

Description: expression of the material weight and energy involved in the productive processes in equivalent C weight by mean of stoichiometric coefficients.

Example: the conversion coefficients that should be used on a livestock farm to obtain the equivalent C weight are 0.27291 for CO_2_, 0.74882 for CH_4_, 0.19998 for CH_4_N_2_O (urea fertiliser), 0.46 for protein [5], 0.70 for fats [5], 0.40 for carbohydrates (C_n_H_2n_O_n_), 0.36 for urine [13], 0.50 for plants or plant derived products (e.g. straw, crop residues, faeces, manure, etc.) calculated from the average molecular formula CH_1.44_O_0.66_ [14], and 0.15–0.20 for the live weight (LW) of the animals, according to the breed [15], 723.2 g/L for oil [16]. For other data, such as C released per kWh of power used, it is necessary to refer to the specific national inventory of the CO_2_ emissions to consider the mix of energy sources used to produce electricity in each country.

Step 8, categorisation of the C fluxes.

Description: Attribution of the C fluxes to the input, output or internal categories.

Example: the inputs on a livestock farm are the purchased seeds, plants, fertilisers, pesticides and medicines that contain C, feeds, litter materials, animals and fuel and the net C exchanged by the plants; the outputs are the sold products (of vegetable or animal origin), the redox of the soil C, organic C dissolved from the soil, gases released during manure storage and spreading, gases of enteric origin, respiratory gases, gases from fuel combustion and power obtained from fossil fuels; the internal fluxes are the products reused in the productive processes on the farms (straw, manure, self-produced feeds, replacing animals, milk of lactating cows, crop residues, etc.).

Step 9, implementation of the balance.

Description: the difference between the inputs and outputs results in a net C change in the system; a positive sign was conventionally adopted for the C inputs, while a negative one was adopted for the outputs. From a mathematical point of view, the fluxes should be summed with their sign to obtain the net carbon balance.

Example: plant growth (+) plus purchased feeds and litter (+) plus fuel (+) plus fertilisers, pesticides and medicines (+) plus soil losses (-) plus animal respiration (-) plus enteric fermentation (-) plus sold animals and/or products of animal origin (-) plus sold plant products (-) plus fuel combustion (-) plus electric energy consumption (-) is equal to the systemic net C balance of a livestock farm.

Step 10, interpretation of the results.

Description: systematic analysis of the results whereby the outcomes (C fluxes and storage or losses from the accumulation sites - plants, animals, soil and air) are classified, quantified, checked, and evaluated by calculating the derived indexes. To this aim, it is important to refer the net C change to one or more functional units (FU). The FU serves as the reference base for all the calculations regarding an impact assessment and allows a comparison of the results of different productive systems with different production organisations or intensities to be made. As it is necessary to make some assumptions, a sensitivity analysis, based on the range of sensitivity of the variables significantly increases the robustness of the results. The interpretation of the results of such a sensitivity analysis can in fact furnish appropriate conclusions and recommendations.

Example: if the balance is positive, the system stores C, if it is negative the system deplete the C stored, but if it is equal to zero the system is in equilibrium. The losses and gains of C from a livestock farm system occur as a result of the exchanges with the atmosphere, that is, the air site. It is possible to evaluate the C efficiency of a system as the ratio between the result of the C balance and the sum of the inputs. If the balance is positive or negative, the system is environmentally efficient or inefficient, respectively; the higher the value, the more efficient or inefficient the system is, depending on the sign of the net C change (+ or -). Finally, it is possible to evaluate the intensity of a system as the ratio of the inputs to the FU adopted to describe the systemic C balance (e.g., hectares of utilised agricultural area, tonnes of LW, tonnes of LW of the slaughtered animals, tonnes of produced milk, etc.).

In the context of this article, the systemic C balance was described in a general manner so that it could be adapted to any type of CIS. It will be up to the experts in a specific production sector to identify the input and output flows, as well as the internal flows, and to select the most appropriate measurement or estimation techniques according to the nature of the flow that has to be quantified. The approach is here provided for a beef cattle production system, albeit only as a guide, because the method was applied to this sector for validation purpose, as described in the subsequent section. However, it is possible to describe the extent of the application of the systemic C balance to other types of integrated biological systems considering that the C exchanges can be solid, liquid or gaseous, and that these flows can concern any biological and non-biological component involved in the process, whether organic, microbiological, vegetal, animal, inorganic or used for energy purposes. The task of fully identifying all these components and the derived flows should once again be entrusted to the experts of each production sector.

## Method validation

The applicability of the systemic C balance was tested on a beef cattle production system. An Italian Consortium of Piemontese breed beef farms (Coalvi) that groups together approximately 1300 livestock farms and raises >130 thousand heads was chosen for this purpose. This application has recently been published in a scientific paper [15] to which the reader can refer. The group of farms in the Consortium was considered as a single system to validate the method, because the feeding systems, diets, and manure management procedures of the considered farms were similar, and the farms are all located in the same climatic area (the Piedmont region, North-West Italy; Latitude N 44.12–45.82, Longitude E 7.12–8.98). The area has an average rainfall of 1050 mm year^−1^ and an annual daily mean temperature of 9.1 °C [17]. The characteristics of these farms ensure homogeneity of the sample but also complexity and integration of the productive processes.

Complexity of the system is ensured by the differentiated productions. These include not only animal production, but also different types of cultivation for the livestock (crops, grasslands, pastures) and/or for the market (crops, orchards, vineyards, woods, etc.). The cultivation list includes 139 different types of cultivation. The herds are managed according to different types of production systems, i.e. whole-cycle system, cow-calf system, growing-finishing system, and mixes of the aforementioned production systems.

The integration of the productive processes carried out by the farms and the integration of the farms are ensured because of the combination of crop, livestock and/or forestry activities on the same farm and because the system of the farms that are members of the Consortium ensures the supply of replaced animals and other products and by-products (e.g. organic fertilisers, hay, etc.).

Thus, the sample was considered suitable to validate the systemic C mass balance as an environmental assessment method that could overcome the limitations of other approaches and to obtain complementary information to that obtained from other environmental assessment methods.

The results of a detailed application of the systemic C balance to the farms in the Consortium have recently been published in the previously cited scientific paper [15]. The C fluxes and storage are shown in Table 1.

**Table 1:** Carbon (C) storage, fluxes (I, input; O, output; N, internal fluxes) and balance of the system presented as the absolute value (total) and relative values (per hectare and per ton of live weight slaughtered, LW).

Category	C (,000 t)	C per hectare (t C ha^-1^)	C per ton LW (t C t^-1^ LW)
C stored in the system sites			
Animals	8.6	0.12	0.23
Plants	109.3	1.55	2.94
Soil	0.0	0.00	0.00
C stored outside the system			
Air	−96.1	−1.36	2.58
C fluxes: animal sector			
(I) Purchased feeds (from outside the system)	5.2	0.07	0.14
(N) Self-produced feeds and litter (from the plant site)	167.2	2.37	4.49
(O) Animal gases (to the air site)	−106.9	−1.51	−2.87
(N) Manure (to the soil site)	−44.7	−0.63	−1.20
(O) Manure (to the air site)	−12.2	−0.17	−0.33
(O) Energy (to the air site)	−0.2	−0.003	−0.005
(O) Animals sold (to outside the system)	−7.3	−0.10	−0.20
C fluxes: plant sector			
(I) Net plant exchange (from the air site)	357.4	5.06	9.60
(I) Fuel (from outside the system)	15.6	0.22	0.42
(N) Crop residues (to the soil site)	−80.9	−1.15	−2.17
(N) Self-produced feeds and litter (to the animal site)	−167.2	−2.37	−4.49
(O) Gas emissions from fuel combustion (to the air site)	−15.6	−0.22	−0.42
(O) Plant products sold (to outside the system)	−27.1	−0.38	−0.73
C fluxes: soil sector			
(N) Crop residues (from the plant site)	80.9	1.15	2.17
(N) Manure (from the animal site)	44.7	0.63	1.20
(I) Fertilisers (from outside the system)	0.8	0.01	0.02
(O) Depletion of organic C (to the air site)	−126.4	−1.79	−3.39
Balance (inputs-outputs)	83.3	1.18	2.24

The systemic C balance resulted to be positive and amounted to 83.3 10^3^ t of C. We calculated that the productive activities of the selected group of beef farms in the Consortium removed 96.1 10^3^ t of C from the atmosphere (air site) over a period of one year, and this amount of C was transferred to plant growth and agricultural products (plant site) and to an increase in LW of the animals (animal site). The rates of the stored C to agricultural and wooded areas and to the LW of the slaughtered animals in one year were 1.18 t ha^−1^ and 2.24 t C t^−1^ LW, respectively. Given the high uncertainty in the measurement of C, a two way sensitivity analysis was carried out to evaluate the uncertainty and the robustness of the obtained results considering the estimated ratio of variation of the C input and output components of the balance. This estimation was introduced using the range of variation of the single components of the C inputs and outputs calculated as weighted means on the most probable range of variation of the single components of the C inputs and outputs, respectively, and on their different contributions (weights) to the final value of these variables. The variations of the C balance components were provided by the Consortium, which collects the annual production data of its members, or were assumed by referring to bibliographic references. The quantifications of the ratio of variation resulted to be ± 10.4 and ± 9.9, for the C inputs and outputs, respectively; both variables were rounded off to a range of variability of ±10 % and were then used to carry out the analysis. The sensitivity analysis applied to these results demonstrated that the C balance was always positive, even for the worst scenario that was hypothesised considering the uncertainty of the balance components. The readers can refer to the previously cited scientific paper [15] for the details on the determination of the ratio of variation of the variables and the results of the sensitivity analysis.

A comparison with a Life Cycle Assessment (LCA) analysis applied to a selection of 10 whole-cycle Piemontese beef farms [18] was carried out to validate and compare the results obtained using the systemic C balance with those from conventional environmental assessment methods. These farms are representative of the most widespread Piemontese beef production and management strategies adopted in North-West Italy and therefore of the group of farms used for the application of the systemic C balance since they are all located in the same area, self-supplied with replaced animals, adopt the same rearing system and management strategies, and obtain the same productions (fattening animals and culled cows). We concluded, through the Global Warming Potential (GWP) calculated with the LCA method that such a system emits 15 kg of CO_2_ equivalent per kg of live weight, which is equivalent to 4.1 kg of C lost versus 2.2 kg of C captured, as calculated with the systemic C balance. Even when nitrous oxide is considered in the LCA calculation of the greenhouse effect, which was not done in the application of the systemic C balance as it did not contain C, it is clear that LCA studies, which do not consider the immobilisation of material in the organic matter that occurs during the production process (e.g. by means of the addition of organic fertilisers to the soil or by the particulate fallout or bacterial fixation of atmospheric N, even for crops not specifically intended for livestock feed production), suffer from some limitations as they adopt a product-based approach. Indeed, the LCA method is not able to evaluate the positive effects of an integrated production system, as it only gives a partial representation of a complex reality.

It is possible to affirm that the results obtained in the present study have led to different conclusions from those that would have been obtained with other LCA analysis, which, in several studies conducted on similar scenarios, led to the conclusion that livestock is a net emitter of greenhouse gases, a result that was confirmed by analysing different and consecutive years [18] and different livestock systems [19]. This is because the main methods that are generally used to assess the environmental impact measure the emissions but neglect the captures or consider them separately, as in the case of the Carbon Footprint [20]. Instead, this study has shown that the examined beef production system, when analysed as an integrated and complex system, can be considered an important C sink, and that it is necessary to reconsider the effect that livestock, and ruminants in particular, have on the global greenhouse effect.

Thus, it appears, on the basis of these preliminary results, that the here presented method is able to describe a complex and integrated system and to highlight the relationship that exists between rearing and agricultural activities in order to characterise their environmental roles.

## Limitations

Despite the positive results that were obtained, it is possible to underline some limitations of the systemic C balance. This method does not analyse the system with regard to the different categories of environmental impact although it does highlight the flows and storage of the materials of the system. From an environmental point of view, the same materials could have different impacts, or different materials could have the same effect, but this does not emerge from the mass balance. However, the method allows one to evaluate whether the system is depleted or enriched by a certain resource. For these reasons, the method was conceived as a supplementary tool to be used, together with other assessment methods, to complete the examination of a CIS by adding a different point of view, without overlapping the other types of evaluation pertaining water use, biodiversity, and/or soil health.

The systemic C balance applied in the aforementioned case study was not able to consider the effects of climate, management or soil characteristics on the actual C fluxes, because it was unable to correlate these variables with the C fluxes. The method provided an evaluation that is useful for comparisons between different production choices and/or different production systems. Climatic, management and pedological forecasting models could eventually be introduced into the balance, with reference to specific climatic areas.

The drawing up of a systemic C balance requires that a great deal of different kinds of raw data are collected from production systems of various complexity. In this paper, the systemic C balance method has been described for operational application at a farm level. Many of the necessary data can be obtained from formulas or coefficients, starting from primary data, as happens for other evaluation methods, such as the LCA method with a Tier 1, 2 or 3 approach. These levels of assessment refer to different levels of detail and complexity in evaluating environmental impacts. However, the use of secondary data in the systemic C balance can also be replaced by data detected directly, under experimental conditions, according to the scope, depth and data quality of the assessment, and the available resources and the required precision can be aligned with the research goals. However, as previously discussed in step 6 of the Method details section, direct measurements do not exclude the risk of incomplete collections, errors or problems, such as those pertaining to the measurement of enteric gases.

The data should be critically evaluated, processed and correctly interpreted by multidisciplinary experts (e.g. agronomists, zootechnicians, soil scientists, agricultural engineers, etc.). In other words, the application of this assessment method is not simple, as it requires a great deal of data, and the results could change according to the species-specific data elaboration process that is carried out to evaluate the material and energy fluxes. This aspect would make a comparison between studies conducted by different research groups difficult, but this is a problem that is common to other assessment methods, such as the LCA method, and depends on the subjective methodological assumptions, e.g. the system boundary, the allocation method or which Tier level is adopted (1, 2 or 3). For these reasons, it should be considered that the C balance offers complementary environmental indexes that can be used to complete the information provided by other assessment methods.

As far as the risk of overlooking the potential negative impacts of intensive practices that are considered part of the balance is concerned, the comparative use of the systemic C balance, through a critical analysis of the results and C fluxes obtained through the use of appropriate indexes, allows an evaluation to be made considering both the positive and negative impacts of different livestock systems and production choices. Despite the limitations of this approach, and which inevitably exist for any assessment method, it appears that the systemic C balance is able to describe a CIS and to highlight the relationship that exists between rearing and agricultural activities in order to characterise their environmental roles.

## Ethics statements

This work did not involve human subjects, animal experiment data, and data collected from social media platforms.

## CRediT authorship contribution statement

**Davide Biagini:** Writing – review & editing, Writing – original draft, Visualization, Validation, Supervision, Project administration, Methodology, Investigation, Formal analysis, Data curation, Conceptualization.

## Declaration of competing interest

The authors declare that they have no known competing financial interests or personal relationships that could have appeared to influence the work reported in this paper.

## Data Availability

Data will be made available on request.
